# Comparative and phylogenetic analysis of the complete chloroplast genome sequences of *Lactuca raddeana* and *Lactuca sativa*

**DOI:** 10.1080/23802359.2021.1911700

**Published:** 2021-04-22

**Authors:** Mei Jiang, Yuning Li, Haimei Chen, Bin Wang, Chang Liu

**Affiliations:** a Key Laboratory of Bioactive Substances and Resource Utilization of Chinese Herbal Medicine from Ministry of Education, Engineering Research Center of Chinese Medicine Resources from Ministry of Education, Institute of Medicinal Plant Development, Chinese Academy of Medical Sciences, Peking Union Medical College, Beijing, P. R. China; b School of Pharmaceutical Sciences, Xiangnan University, Chenzhou, P. R. China

**Keywords:** *Lactuca raddeana*, Chloroplast genome, DNA barcode, Phylogeny

## Abstract

*Lactuca raddeana* is a biennial plant of *the Lactuca* genus belonging to the Asteraceae family. The classification of *Lactuca* is controversial, and no consistent conclusions have been reached based on the analysis of morphological characters and different molecular markers. Here, we sequenced and assembled the complete chloroplast genome of *L. raddeana*. This genome has a total length of 152,339 bp, a conservative quartile structure that is composed of a large single-copy (LSC) region of 83,976 bp, a small-copy (SSC) region of 18,521 bp, and a pair of inverted repeats (IRs) of 24,921 bp. The genome contains 112 unique genes, including 79 protein-coding, four rRNA, and 29 tRNA genes. Repeat analysis obtained 17 microsatellite, 16 tandem, and 17 interspersed repeats. Comparison of sequence divergence between *L. raddeana* and *L. sativa* found the intergenic spacer *trn*C-*pet*N exhibited the highest degree of variation. Three phylogenetic trees based on the 72 shared protein, *mat*K gene, and *rbc*L gene sequences showed that *L. raddeana* and *L. sativa* are clustered together. The acquisition and comparative analysis of the chloroplast genome provide a valuable resource for the taxonomic and phylogenetic studies of *Lactuca*.

## Introduction

*Lactuca raddeana* belongs to the genus *Lactuca*, family Asteraceae, distributed in China, Russia, North Korea, Japan, and South Korea. It grows in forests, forest margins, thickets, moist areas on mountain slopes, mountain valleys, fields, and trailside (iPlant 2020a). The *Lactuca* genus includes at least 50 species, distributed worldwide, but mainly in temperate Eurasia (iPlant 2020b). For *L. raddeana*, it is found that its chemical component lactuside B improved ischemic brain injury by reducing aquaporin-4 and caspase-3 mRNA expression in the hippocampus and striatum three and regulating the expression of BCL-2 and BAX mRNA (Li et al. 2011). However, the effects of other species from this genus remained to be discovered. Clarification of the taxonomic classification of *L. raddeana* is critical for the future development of new drugs based on *L. raddeana*.

The classification of *Lactuca* is controversial. *L. raddeana* has several alias names, including *Pterocypsela elata* (Hemsl.) Shih, *Pterocypsela raddeana* (Maxim.) Shih, *Lactuca vaniotii* H. Lév., *Lactuca alliariifolia* H. Lév. et Vaniot, *Lactuca elata* Hemsl., *Lactuca raddeana* Maxim. var. elata (Hemsl.) Kitam. and *Prenanthes hieraciifoli*a H. Léveillé (iPlant, 2020a), reflecting the difficulty in determining its taxonomy classification using morphological characters. More recently, the nuclear rDNA ITS sequences and five chloroplast DNA markers had been used to delineate the phylogeny of *Lactuca* species. Unfortunately, inconsistent results were obtained using these markers (Wang et al. 2013). The phylogeny of *Lactuca* was analyzed based on 31 chloroplast genomes (LSC + SSC + IR), nuclear rDNA, and ITS sequences, indicating that there are at least four main groups (Wei 2016). However, these species cover just 36% of the *Lactuca*, and there are still some differences among the three trees. Thus, more phylogenetic analysis based on chloroplast genomes is needed to study the phylogeny of *Lactuca* furtherly.

The chloroplast is an essential organelle in plant cells and capable of photosynthesis in the presence of light. The complete chloroplast genome provides a rich resource for accurate identification of plant species and determining their phylogenetic relationships (Daniell et al. 2016). Chloroplast genome encodes many key proteins playing essential roles in photosynthesis and other metabolic processes (Keeling 2004). The structure of the chloroplast genome is highly conserved and usually consists of four parts with a large single-copy (LSC) region, a small single-copy (SSC) region, and two inverted repeats (IRs) (Palmer 1991). The chloroplast genome sequence can be divided into the protein-coding region and the non-coding region. The coding region has a slow evolution rate and is suitable for phylogenetic analysis of higher taxonomic levels such as order and family (Li et al. 2012). The non-coding region evolves faster and has more variation information, which is more suitable for phylogenetic analysis of lower taxonomic levels such as genus and species (Shaw et al. 2007).

Previously, the partial chloroplast genome sequence of *Lactuca raddeana* has been reported, which has 127,660 bp containing an LSC, an SSC, and one IR region (Wei 2016). However, this genome sequence was not publicly available. To address this problem, we sequenced, assembled, and annotated the complete chloroplast genome sequence of *L. raddeana* and compared it with the chloroplast genomes of *L. sativa.*

## Material and methods

### Plant material, DNA extraction, and sequencing

We collected fresh leaf samples from the Central China Medicinal Botanical Garden, EnShi, China (Geospatial coordinates: N30.178176, E107.745725) and identified them as *L. raddeana* by Professor Jinwen You. We kept the sample in the −80° refrigerator until use. We extracted the genomic DNA with plant genomic DNA kit (Tiangen Biotech, China) and constructed the DNA library with 1 ug DNA using the library preparation kit (New England BioLabs. America). We sequenced the library using the Hiseq 2500 platform (Illumina, America). A total of 26,321,582 paired-end reads (2 × 150 bp) were obtained. Clean data were obtained by removing low-quality sequences: the percentage of bases with a quality value of Q < 19 is more than 50%, and the percentage of ‘N’ is more than 5%.

### Genome assembly and annotation

We assembled the clean sequence data into a chloroplast genome by NOVOPlasty (v. 2.7.2) (Dierckxsens et al. 2017) with the k-mer length of 39 bp and the sequence of *rbc*L from *Arabidopsis thaliana* as the seed. We manually examined the assembly by mapping all raw reads to the assembled genome sequence using Bowtie2 (v.2.0.1) (Langmead et al. 2009) under the default settings. Complete coverage and an even sequencing depth across the assembly would support the correctness of the assembly. The annotation of the chloroplast genome was initially performed using CpGAVAS2 (Shi et al. 2019), and then the annotations with problems were edited using Apollo (Misra and Harris 2006). The genome sequence and annotations have been deposited in GenBank with accession number MN402448.

### General characteristics and repeat analysis

The codon usage and repeat analysis were analyzed using CpGAVAS2. Notably, we identified the microsatellite repeats with MISA software (Beier et al. 2017). And the cutoff for the numbers of units for mono-, di-, tri-, tetra-, Penta-, and hexanucleotides were 10, 6, 5, 5, 5, and 5, respectively. We identified the tandem repeats by using TRF software (Benson 1999) with the size of repeat unit > = 7. Then, we identified the interspersed repeats with VMATCH software (Kurtz et al. 2001). Lastly, we calculated both GC contents and codon usage using the program Cusp from EMBOSS (v6.3.1) (Rice et al. 2000).

### Comparative analysis of the chloroplast genomes

The comparative analysis between *L. raddeana* and the *L. sativa* was conducted using the mVISTA program (Frazer et al. 2004) in a Shuffle-LAGAN mode with default parameters. The complete chloroplast genome of *L. sativa* (Accession number: NC_007578) was obtained from Genbank. And the annotated *L. raddeana* genome was used as the reference. The IR boundary was analyzed and visualized by using the IRscope tool (Amiryousefi et al. 2018).

### Phylogenetic analysis

We constructed the phylogenetic tree between the *L. raddeana* and other fourteen closely related species obtained from Genbank (Table S4), *L. sativa*, *Sonchus acaulis*, *Sonchus boulosii*, *Sonchus canariensis*, *Sonchus webbii*, *Taraxacum amplum*, *Taraxacum brevicorniculatum*, *Taraxacum kok-saghyz*, *Taraxacum mongolicum*, *Taraxacum obtusifrons*, *Taraxacum officinale*, *Taraxacum platycarpum*, *Youngia japonica*, and *Youngia denticulata*. *Helianthus annuus* and *Ageratina adenophora* were set as outgroups. A total of 72 shared proteins present among the sixteen chloroplast genomes were detected and extracted (ATPA, ATPB, ATPE, ATPF, ATPH, ATPI, CCSA, CEMA, CLPP, MATK, NDHA, NDHB, NDHC, NDHD, NDHE, NDHF, NDHG, NDHH, NDHI, NDHJ, NDHK, PETA, PETB, PETD, PETG, PETL, PETN, PSAA, PSAB, PSAC, PSAI, PSAJ, PSBA, PSBB, PSBC, PSBD, PSBE, PSBF, PSBH, PSBJ, PSBK, PSBM, PSBN, PSBT, RBCL, RPL14, RPL16, RPL2, RPL20, RPL22, RPL23, RPL32, RPL33, RPL36, RPOA, RPOB, RPOC1, RPOC2, RPS11, RPS12, RPS14, RPS15, RPS16, RPS18, RPS19, RPS2, RPS3, RPS4, RPS7, RPS8, YCF3, YCF4). These protein sequences were subjected to multiple sequence alignment using CLUSTALW2 (v2.0.12) (Thompson et al. 2002) with the option ‘-gapopen = 10 -gapext = 2 -output = phylip’. The phylogenetic tree was then constructed using the Maximum Likelihood method implemented in RaxML (v8.2.4) (Stamatakis 2014) with the option ‘raxmlHPC-PTHREADS-SSE3 -f a -N 1000 -m PROTGAMMACPREV -x 551314260 -p 551314260 -o A_thaliana, N_tabacum -T 20’.

## Results

### General features of the chloroplast genome

**Figure 1.**
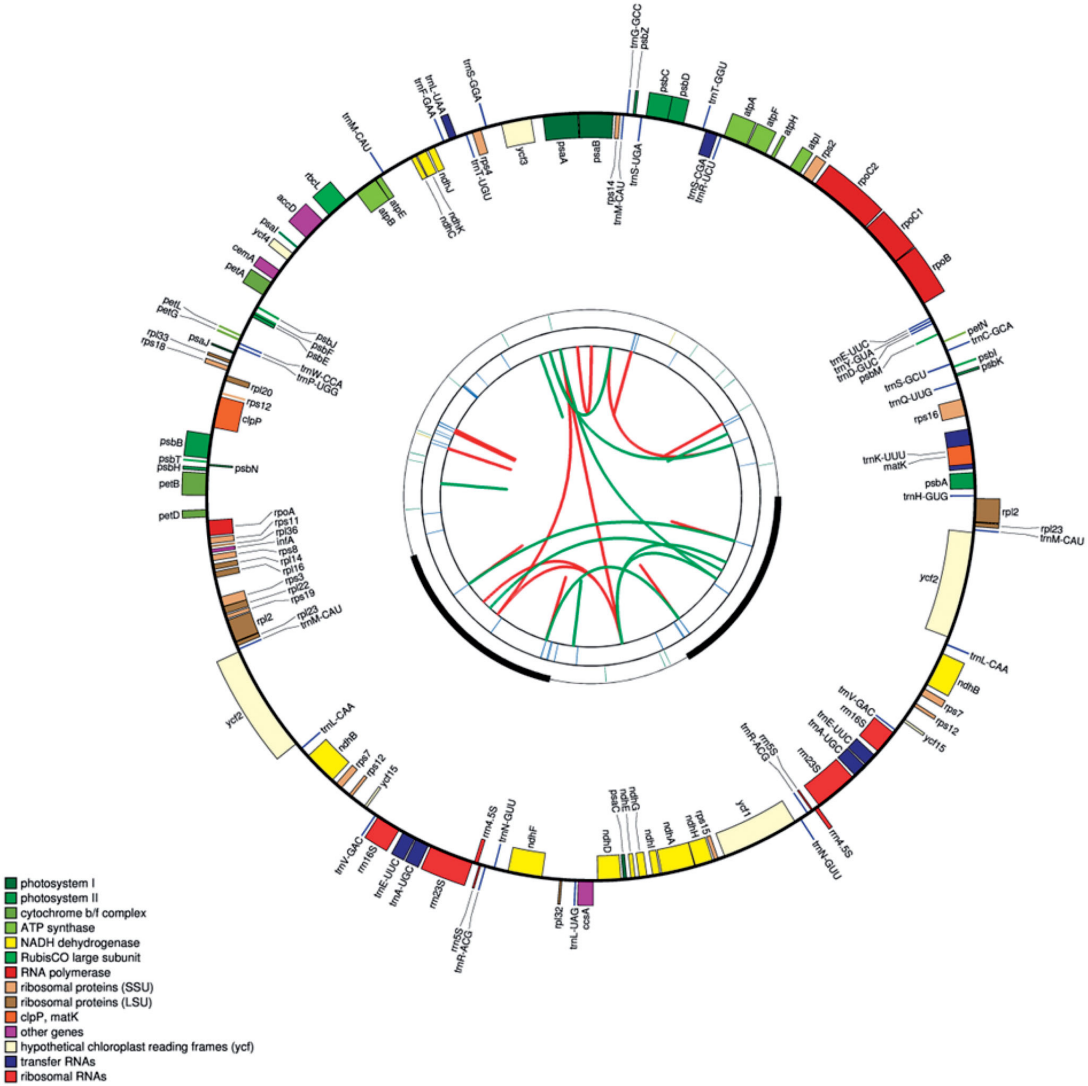
The schematic representation of the chloroplast genome of *L. raddeana* was created by using CPGAVAS2. The map contains four rings. From the center going outward, the first circle shows the scattered forward and reverse repeats connected with red and green arcs, respectively. The next circle shows the tandem repeats marked with short bars. The third circle shows the microsatellite sequences identified. The fourth circle shows the gene structure of the chloroplast genome. The genes were colored based on their functional categories, which are shown in the left corner.

The chloroplast genome of *L. raddeana* is 152,339 bp in size with an LSC of 83,976 bp, an SSC of 18,521 bp, and two IRs of 24,921 bp each (Figure 1). The chloroplast genome encodes 130 genes, of which 112 are unique genes, including 79 protein-coding genes, four ribosome RNA genes, and 29 transfer RNA genes (Table 1). Among them, there are 7 genes (*rps*16, *rpo*C1, *atp*F, *pet*B, *rpl*2, *ndh*B, and *ndh*A) contains 1 intron, 2 genes (*ycf*3 and *clp*P) contain 2 introns. 6 tRNAs (*trn*K-UUU, *trn*S-CGA, *trn*L-UAA, *trn*C-ACA, *trn*E-UUC, and *trn*A-UGC) contain 1 intron (Table S1). The length of the protein-coding regions, the rRNA genes, and the tRNA genes in the chloroplast genome of the *L. raddeana* is 78,288 bp, 9,536 bp, and 2,727 bp, accounting for 51.39%, 6.26%, and 1.79% of the genome length, respectively. The chloroplast genome's non-coding region mainly includes introns and intergenic spacers, and its length accounts for 40.56% of the genome length.

**Table 1.** Gene composition in the chloroplast genome of *L. raddeana*.

Category of genes	Group of genes	Name of genes
	rRNA	*rrn*16S^2^, *rrn*23S^2^, *rrn*5S^2^, *rrn4.*5S^2^
	tRNA	29 trna genes (6 contain an intron)
Photosynthesis	Subunits of ATP synthase	*atp*A, *atp*B, *atp*E, *atp*F, *atp*H, *atp*I
	Subunits of photosystem II	*psb*A, *psb*B, *psb*C, *psb*D, *psb*E, *psb*F, *psb*I, *psb*J, *psb*K, *psb*M, *psb*N, *psb*T, *psb*Z, *ycf*3
	Subunits of NADH-dehydrogenase	*ndh*A, *ndh*B^2^, *ndh*C, *ndh*D, *ndh*E, *ndh*F, *ndh*G, *ndh*H, *ndh*I, *ndh*J, *ndh*K
	Subunits of cytochrome b/f complex	*pet*A, *pet*B, *pet*D, *pet*G, *pet*L, *pet*N
	Subunits of photosystem I	*psa*A, *psa*B, *psa*C, *psa*I, *psa*J
	Subunit of rubisco	*rbc*L
Self replication	Large subunit of ribosome	*rpl*14, *rpl*16, *rpl*2^2^, *rpl*20, *rpl*22, *rpl*23^2^, *rpl*32, *rpl*33, *rpl*36
	DNA dependent RNA polymerase	*rpo*A, *rpo*B, *rpo*C1, *rpo*C2
	Small subunit of ribosome	*rps*11, *rps*12^2^, *rps*14, *rps*15, *rps*16, *rps*18, *rps*19, *rps*2, *rps*3, *rps*4, *rps*7^2^, *rps*8
Other genes	Subunit of Acetyl-CoA-carboxylase	*acc*D
	c-type cytochrom synthesis gene	*ccs*A
	Envelop membRNAe protein	*cem*A
	Protease	*clp*P
	TRNAslational initiation factor	*inf*A
	Maturase	*mat*K
Unknown	Conserves open reading frames	*ycf*1, *ycf*15^2^, *ycf*2^2^, *ycf*4

The ‘2’ symbol after gene name reflect the genes located on the IR regions.

The whole genome's GC content is 37.69%, of which the protein-coding regions, the rRNA genes, and the tRNA genes are 38.02%, 54.55%, and 53.12%, respectively. The GC contents for the first, second, and third positions of the codons are 45.58%, 38.26%, and 30.22%, respectively, showing a higher A/T bias within the protein-coding regions at the third codon position. In terms of codon usage, 26,096 codons encoding 79 protein-coding genes were identified in the complete genome (Table S2). The most common codon, AUU, codes for the amino acid Isoleucine (an abbreviation I) and was found 1,056 times.

**Table 2.** Microsatellite repeats in the chloroplast genome of *L. raddeana*.

SSR type	Structure of SSRs	Size (bp)	Start	End	Region (gene names)
p1	(A)10	10	4408	4417	IGS (matK-rps16)
p1	(T)15	15	12,680	12,694	IGS (trnE-UUC- rpoB)
p1	(A)11	11	16,599	16,609	Intron (rpoC1)
p1	(A)10	10	18,409	18,418	Exon (rpoC1)
p1	(T)10	10	23,213	23,222	IGS (rpoC2- rps2)
p2	(AT)6	12	26,596	26,607	IGS (atpH- atpF)
p1	(A)10	10	43,666	43,675	Intron (ycf3)
p1	(T)10	10	50,222	50,231	IGS (ndhC- trnM-CAU)
p1	(T)10	10	59,176	59,185	IGS (psaI- ycf4)
p1	(T)10	10	67,305	67,314	IGS (psaJ- rpl33)
p2	(TA)7	14	67,647	67,660	IGS (rpl33- rps18)
p1	(T)11	11	70,358	70,368	Exon (clpP)
p1	(T)10	10	71,329	71,338	Intron (clpP)
p1	(T)10	10	77,876	77,885	Exon (rpoA)
p1	(A)10	10	116,247	116,256	IGS (ndhD- psaC)
p1	(T)10	10	124,260	124,269	Exon (ycf1)
p1	(T)10	10	124,985	124,994	Exon (ycf1)

p1: perfect repeat of mononulceotide; p2: perfect repeat of dinucleotides. The structure of SSRs is presented as repeat units surrounded with parenthesis, followed with the number of repeat units.

### Repeat analysis

The types and numbers of repeated sequences might provide important information regarding genome evolution. There are three types of repeats. The simple sequence repeats (SSRs), also referred to as microsatellite repeats, consist of multiple copies of small repeat units (size <= 6 bp) concatenated together. They are abundant in the angiosperm genomes and often used as molecular markers because they mutate rapidly at the interspecies and intraspecies levels (Lu et al. 2005; Tanaka et al. 2017). In the chloroplast genome of *L. raddeana*, the SSRs include 15 A/T and 2 AT/AT (Table 2). These numbers are rather small compared to those found in other chloroplast genomes (data not shown). The SSRs were found to distribute across the intergenic spacers (IGS), intron regions, and exon regions. And numbers of SSRs falling into these regions are 9, 3, and 3, respectively.

The second type of repeats is the tandem repeat consisting of multiple copies of repeat units (size > 6 bp) concatenated together. A total of 26 tandem repeats were found in the chloroplast genome of *L. raddeana*, of which the most extended repeat unit is 32 bp (Table 3). And the number of repeat units is mostly 2. The average matching rate of repeat unit sequences is 98%. Seventeen of the tandem repeats possessed the highest possible matching rate of 100%. Besides, four tandem repeats are present in the IR regions and located in the IGS (*trn*N-GUU-*trn*R-ACG and *trn*N-GUU-*trn*R-ACG) and exonic (*rrn*4.5S and *ycf*2) regions.

**Table 3.** Tandem repeats in the chloroplast genome of *L. raddeana*.

Start-end sites of tandem repeats	Repeat units			Region (gene name)
	Size (bp)	Percent of match	Sequence (copy number)	
4871–4908	19	100	TAAAGAACTTCGATTCCTT (2)	IGS (*mat*K-*rps*16)
8639–8682	21	100	TATGTTACATTACATATCAAG (2)	IGS (*trn*S-GCU)
11,771–11,808	19	94	ACCAATGGACCATAGGGGC (2)	Exon (*trn*E-UUC)
12,249–12,305	28	100	ATTGAGCCCTAAATAATACTGTCAATTG (2)	IGS (*trn*E-UUC-*rpo*B)
31,073–31,106	17	94	GTTAGAGTATATTAGTT (2)	IGS (*trn*T-GGU-*psb*D)
31,492–31,531	19	100	ATAATATTTCTATATTTAG (2)	IGS (*trn*T-GGU-*psb*D)
56,199–56,248	25	92	ATTTGAGTTTCAGGCAATGGATACT (2)	Exon (*rbc*L)
58,617–58,646	15	100	TTCAATATATTCAAT (2)	IGS (*acc*D-*psa*I)
58,620–58,655	15	90	AATATATTCAATATA (2)	IGS (*acc*D-*psa*I)
58,743–58,790	24	100	AATAATATCTCATCCATATCCTTT (2)	IGS (*acc*D-*psa*I)
58,879–58,914	18	100	TATTAAGTAATAATAATA (2)	IGS (*acc*D-*psa*I)
65,047–65,095	24	100	AATAGAAATGACTAGTCTTAGAAT (2)	IGS (*psb*E-*pet*L)
65,473–65,528	27	100	TTTTATACAGTTGTGACTTTATCCTTG (2)	IGS (*psb*E-*pet*L)
66,338–66,369	16	100	GTCCAATGTGAATTGA (2)	IGS (*trn*W-CCA-*trn*P-UGG)
67,780–67,820	21	95	TAAATCCAAGCGAACTTTTCT (2)	Exon (*rps*18)
68,264–68,319	26	96	TATTCTTATCATATTCTATTCATTTT (2)	IGS (*rps*18-*rpl*20)
81,570–81,611	21	100	TTTGTATAGATAACTAAATAT (2)	IGS (*rpl*16-*rps*3)
91,223–91,282	18	100	CGATATTGATGCTAGTGA (3)	Exon (*ycf*2)
106,912–106,973	32	93	CATTGTTCAACTCTTTAACAACACGAAAAAAC (2)	Exon (*rrn*4.5S)
107,809–107,839	15	100	AGTAGCATAACAAAA (2)	IGS (*trn*R-ACG- *trn*N-GUU)
107,947–107,984	18	100	TAATTGGATAGTTGTAAA (2)	IGS (*trn*R-ACG- *trn*N-GUU)
112,130–112,177	22	96	TTATATTATAGACAAATATTAT(2)	IGS (*ndh*F-*rpl*32)
128,332–128,369	18	100	TATTTACAACTATCCAAT (2)	IGS (*trn*N-GUU-*trn*R-ACG)
128,477–128,507	15	100	TTTTTGTTATGCTAC (2)	IGS (*trn*N-GUU-*trn*R-ACG)
129,343–129,404	32	93	TTTTTCATGTTGTCAAAGAGTTGAACAATGGT (2)	Exon (*rrn*4.5S)
145,034–145,093	18	100	ATATCGTCACTAGCATCA (3)	Exon (*ycf*2)

The third type of repeats is the interspersed repeat. It differs from the tandem repeat in the organization. Remarkably, the repeat units are distributed in a scattered and nonadjacent manner within the genome. We identified two tandem repeats in the chloroplast genome, 9 palindromic repeats, and 16 direct repeats (Table S3). The repeats sequences of three types in the chloroplast genome of *L. raddeana* will be invaluable in developing molecular markers.

### Comparative genomic analysis

**Figure 2.**
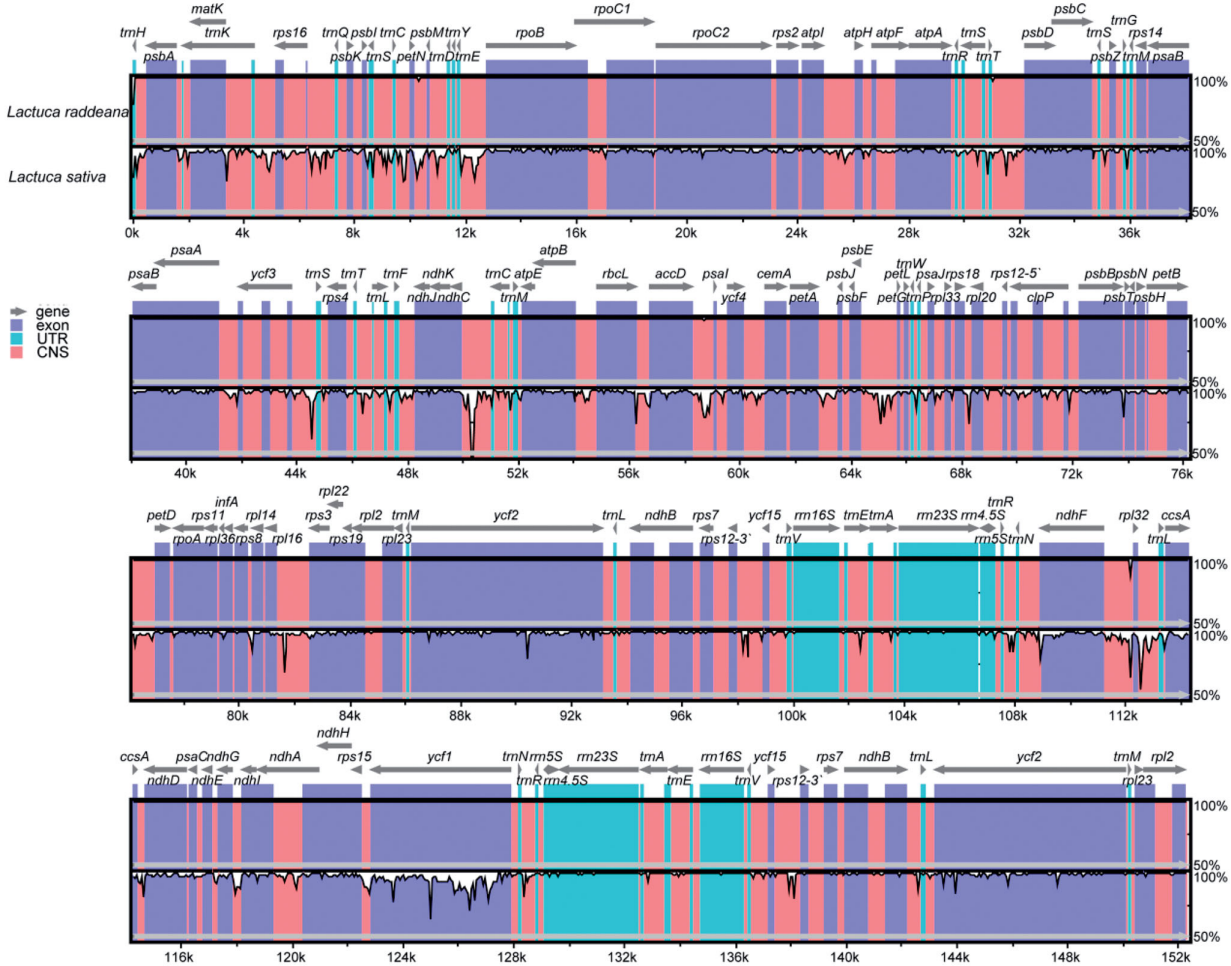
Comparison of the chloroplast genomes of *L. raddeana* and *L. sativa* by using mVASTA. The gray line and arrow indicated the orientation of genes. The coloring of the different conserved regions corresponds to the annotation of the region. The genes were represented as gray arrows on the top of the alignment. Other regions are labeled with different colors. The pink regions are ‘Conserved Non-Coding Sequences’ (CNS), the dark blue regions are exons, and the light-blue regions are ‘Untranslated regions’ (UTR). The Y-scale represents the percent identity ranging from 50% to 100%.

To investigate the similarities and divergence between *L. raddeana* and other *Lactuca* plants' chloroplast genomes, we compared the sequence divergence between the chloroplast genomes of *L. raddeana* and the *L. sativa* (Figure 2). In general, the two genomes are similar, in which the protein-coding regions are more conserved than the non-coding regions. Specifically, the *ycf*1 genes from the two species are highly variable, consistent with the previous finding that the *ycf*1 gene exhibited a rapid evolution rate in many species. For the non-coding regions, seven regions have a higher degree of divergence, with the percentage of similarity being less than 75%. These include IGS(*trn*C-*pet*N), IGS(*trn*E-*rpo*B), IGS(*ycf*3-*trn*S), IGS(*ndh*C-*trn*M), IGS(*psb*F-*pet*G), IGS(*rpl*16-*rps*3), IGS(*rpl*32-*trn*L).

**Figure 3.**
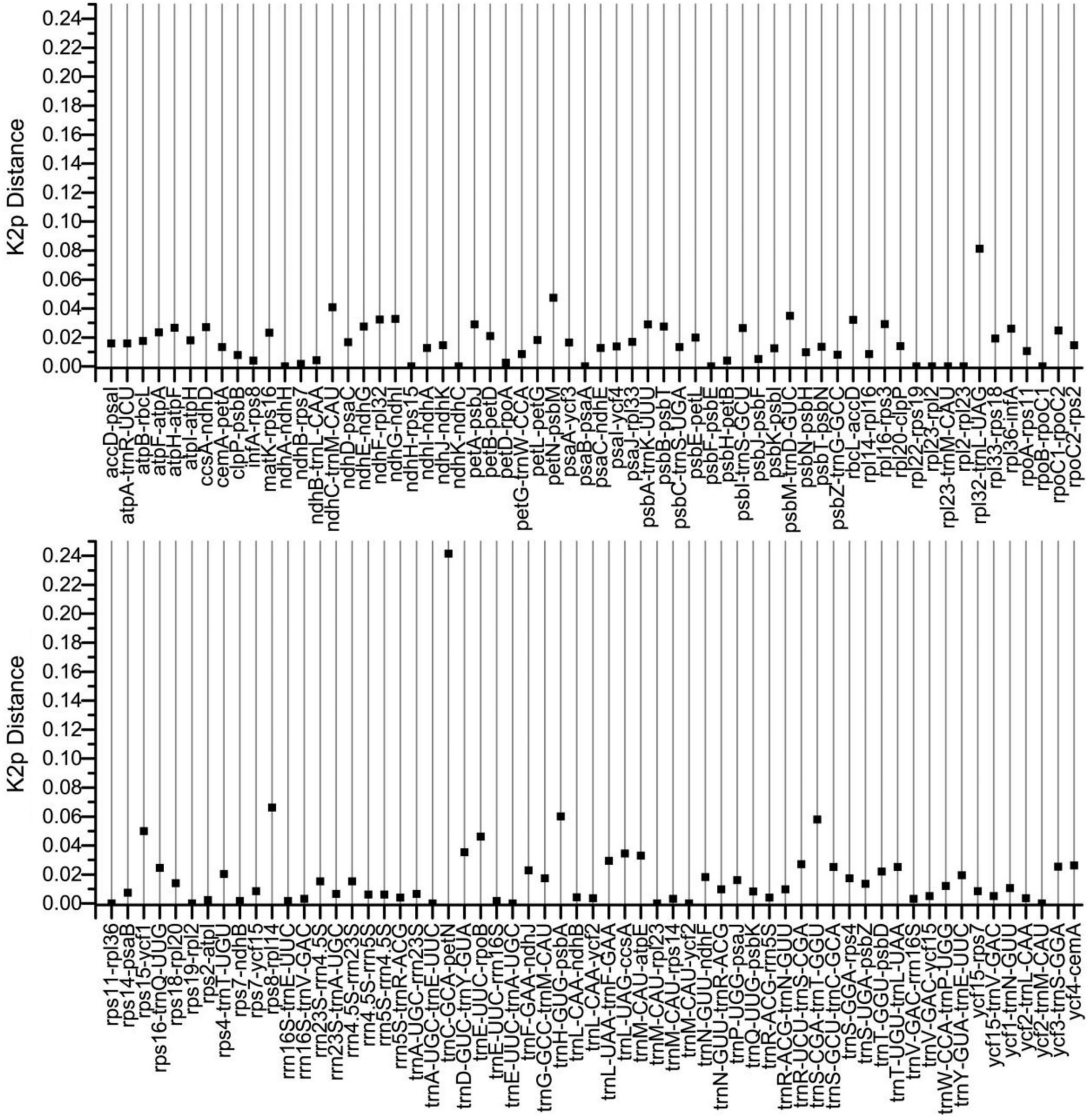
K2p distances between the IGS regions of *L. raddeana* and *L. sativa*.

The distances of intergenic spacers between the *L. raddeana* and *L. sativa* were conducted using the Kimura 2-parameter (K2p) model (Figure 3). The K2p distances range from 0.00-0.242, with an average of 0.018. The seventeen IGS regions: *ndh*A-*ndh*H, *ndh*H-*rps*15, *ndh*K-*ndh*C, *psa*B-*psa*A, *psb*F-*psb*E, *rpl*22-*rps*19, *rpl*23-*rpl*2, *rpl*23-*trn*M, *rpl*2-*rpl*23, *rpo*B-*rpo*C1, *rps*11-*rpl*36, *rps*19-*rpl*2, *trn*A-*trn*E, *trn*E-*trn*A, *trn*M-*rpl*23, *trn*M -*ycf*2, *ycf*2-*trn*M, exhibiting 100% similarity with the K2p values of 0. These IGS regions are conserved and have a length range from 1 bp to165bp. Besides, the IGS(*trnC*-*petN*) has the largest K2p values of 0.242, which indicated a faster evolution rate than other regions. The length of IGS(*trn*C-*pet*N) varies greatly, 534 bp for *L. raddeana* and 831 bp for *L. sativa*. In summary, from the highly variable region, it is expected to develop new DNA barcodes to discriminate different species of *Lactuca*.

### Structure analysis of the IR boundaries

**Figure 4.**
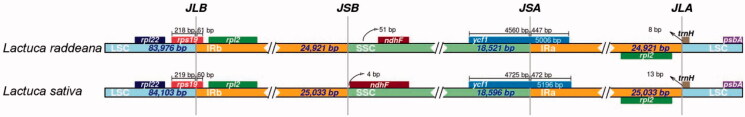
LSC, SSC, and IR regions' boundaries in the chloroplast genomes of *L. raddeana* and *L. sativa*. The genes on the positive and negative strands are presented above and below the tracks, respectively. The JLB, JSB, JSA, and JLA represent junction sites of IRb and LSC, IRb and SSC, SSC and IRa, and IRa and LSC, respectively.

The contraction and expansion of IR boundaries are often related to species evolution and directly affect the chloroplast genome's size. Previous studies have shown that the length and boundary of the IR region are variable in different species. Here the IR boundaries of *L. raddeana* and *L. sativa* chloroplast genomes were analyzed, showing that they have similar boundary structures (Figure 4). The LSC-IRb and SSC-IRa boundaries are located in the protein-coding regions, the *rps*19, and the *ycf*1 genes. In contrast, the IRb-SSC and IRa-LSC boundaries are located in the IGS, the IGS(*trn*N -*ndh*F), and IGS(*rpl*2*-trn*H), respectively. It should be pointed out that not all genes located in the IR regions are duplicated. For example, there is only one ycf1 gene since part of the *ycf*1 gene is located in the IRa region, and the other part is in the SSC region. Compared with other angiosperms, the *Lactuca* genus' IR boundaries were relatively conservative and had no significant contraction and expansion (Chen et al. 2018).

### Phylogenetic analysis

**Figure 5.**
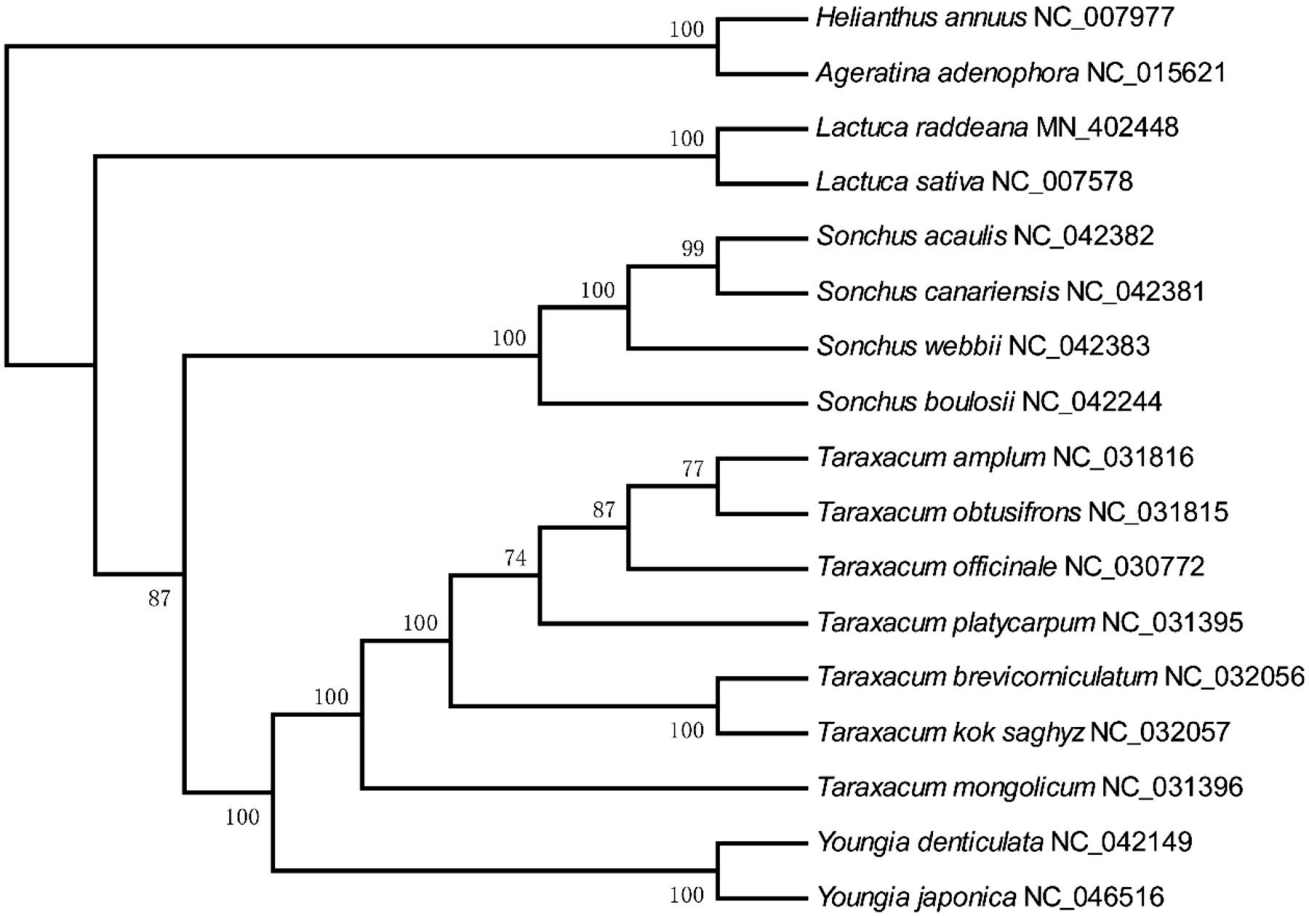
The phylogenetic tree of species from Cichorieae constructed using the maximum likelihood (ML) method using the 72 shared proteins present among the seventeen chloroplast genomes. The number above each node represents are the bootstrap value.

The chloroplast genome provides a valuable resource for the phylogenetic and taxonomic studies of the angiosperm (Guo et al. 2017). We obtained fifteen complete chloroplast genome sequences to explore the phylogenetic relationships among species from Cichorieae. The sequences of 72 shared proteins were used to construct the phylogenetic tree. In general, the species belong to the same genus were clustered together (Figure 5). The genus *Taraxacum*, Sonchus, and Youngia were clustered together, and the *Lactuca* species formed a single phylogenetic cluster. Some taxonomists classified the *L. raddeana* as a species of *Pterocypsela* Shih's genus, and the classification of the genus *Pterocypsela* Shih and *Lactuca* themselves is highly controversial (Wang et al. 2013). In this study, *L. raddeana* and *L. sativa* were clustered together with 100 bootstrap value, which supports *L. raddeana* belongs to the *Lactuca* genus.

## Discussion

In this study, we sequenced and analyzed the complete chloroplast genome of *L. raddeana*. Specifically, we extracted and sequenced the total DNA from the leaves of *L. raddeana*, assembled and annotated a complete chloroplast genome. Then we analyzed the general features of the chloroplast genome and the three kinds of repeat sequences, compared the genomic divergence and IR boundaries between the *L. raddeana* and *L. sativa*, and conducted phylogenetic analyses of *L. raddeana* and several closely related species from the Cichorieae. The present study provides a high-quality reference chloroplast genome sequence for the *L. raddeana*, which can be used for phylogenetic analyses of *Lactuca* species.

The *Lactuca* belong to the tribe Cichorieae, family Asteraceae. Due to the ambiguous definition of the genus, the number of species given in the literature varies greatly. The traits between *Lactuca* and its related genus are complex and have a serious crossover. There are two main types in the classification of Lactuca according to morphological characteristics. The first type, the *Cicerbita Wallr.*, *Mycelis Cass.*, *Mulgedium Cass.*, and *Pterocypsela*, is combined as the *Lactuca* genus section (Lebeda et al. 1999; Doležalová et al. 2002). The second type, the *Cicerbita Wallr.*, *Mycelis Cass.*, *Mulgedium Cass*., and *Pterocypsela*, is considered an independent genus (Chu 1988).

The *Lactuca raddeana* of this study is also named *Pterocypsela raddeana* in the same literature (Wang et al. 2013). This study constructed a phylogenetic tree based on the shared protein sequence, showing that *L. raddeana* and *L. sativa* are clustered together with 100 bootstrap values. Previous studies based on different molecular sequences have shown different phylogenetic results. The phylogenetic analysis based on the entire chloroplast genome sequence without one of the IR regions showed that the *Pterocypsela* group, including *L. raddeana*, was clustered together with those of the crop group *L. sativa* (Wei 2016). The phylogenetic analysis based on *pet*D gene, IGS(*psb*A-*trn*H), IGS(*trn*L-UAA-*trn*F), IGS(*rpl*32-*trn*L-UAG), and IGS(*trn*Q-UUG-*rps*16) showed that the genus *Pterocypsela* including *L. raddeana* and *Lactuca* formed two separate branches (Wang et al. 2013). The phylogenetic tree based on ITS region indicated that the *Pterocypsela* (including *L. raddeana*) and some of the *Lactuca* species (including *L. sativa*) were on one branch (Wang et al. 2013).

What’s more, the *L. raddeana* are not clustered together with *L. sativa* according to the tree constructed using the *ndh*F and *trn*L genes, but on the same branch with other *Lactuca* species ^(^Wei et al. 2017). In summary, the study based on part and complete chloroplast genome supported the notion that the *Lactuca raddeana* (*Pterocypsela raddeana*) belongs to the *Lactuca* genus. However, the current study cannot resolve the classification of all the *Lactuca* species due to limited numbers of chloroplast genomes available. More chloroplast genome sequences are needed to determine the phylogeny of these species ultimately.

## Authors’ contributions

CL conceived the study; MJ collected samples of *L. raddeana*, extracted DNA for next-generation sequencing, assembled and validated the genome; YNL performed data analysis, and drafted the manuscript. BW, HMC, and CL critically reviewed the manuscript. All authors have read and agreed on the contents of the manuscript.

## Data Availability

The data that support the findings of this study are openly available in NCBI (National Center for Biotechnology Information). The accession number of the annotated chloroplast genome is MN402448 (https://www.ncbi.nlm.nih.gov/nuccore/MN402448). The accession number of the raw sequence data is PRJNA688122. The sample has been deposited in the Herbarium of the Institute of Medicinal Plant Development in Beijing, China, with the accession number: implad201808023.
